# Later cART Initiation in Migrant Men from Sub-Saharan Africa without Advanced HIV Disease in France

**DOI:** 10.1371/journal.pone.0118492

**Published:** 2015-03-03

**Authors:** Laure-Amélie de Monteynard, Rosemary Dray-Spira, Pierre de Truchis, Sophie Grabar, Odile Launay, Jean-Luc Meynard, Marie-Aude Khuong-Josses, Jacques Gilquin, David Rey, Anne Simon, Juliette Pavie, Aba Mahamat, Sophie Matheron, Dominique Costagliola, Sophie Abgrall, on behalf of the French Hospital Database on HIV

**Affiliations:** 1 Sorbonne Universités, UPMC Université Paris 06, UMR_S 1136, Institut Pierre Louis d’Epidémiologie et de Santé Publique, Paris, France; 2 INSERM, UMR_S 1136, Institut Pierre Louis d’Epidémiologie et de Santé Publique, Paris, France; 3 AP-HP, Hôpitaux Universitaires Paris-Ile de France-Ouest, Hôpital Raymond-Poincaré, Département de Médecine Aigüe Spécialisée, Garches, France; 4 Université Paris Descartes, Sorbonne Paris cité, Paris, France; 5 AP-HP, Groupe Hospitalier Cochin Broca Hôtel-Dieu, Unité de Biostatistique et Epidémiologie, Paris, France; 6 AP-HP, Hôpital Cochin, Paris, France; 7 AP-HP, Hôpital Saint Antoine, Service des Maladies Infectieuses et Tropicales, Paris, France; 8 Centre Hospitalier Saint-Denis, Hôpital Delafontaine, Service des Maladies Infectieuses et Tropicales, Saint-Denis, France; 9 AP-HP, Hôpital Hôtel-Dieu, Unité d’immunoinfectiologie, Paris, France; 10 Hôpitaux Universitaire Strasbourg, Centre de Soins de l’Infection par le VIH, Strasbourg, France; 11 AP-HP, Groupe Hospitalier Pitié-Salpêtrière, Département de médecine interne, Paris, France; 12 AP-HP, Hôpital Européen Georges Pompidou, Service d’immunologie clinique, Paris, France; 13 Centre Hospitalier Andrée Rosemon, Service des Maladies Infectieuses et Tropicales, Cayenne, France; 14 Université Paris Diderot, Sorbonne Paris cité, Paris, France; 15 AP-HP, Hôpital Bichat-Claude Bernard, Service des Maladies Infectieuses et Tropicales, Paris, France; 16 AP-HP, Hôpital Avicenne, Service des Maladies Infectieuses et Tropicales, Bobigny, France; University of Pittsburgh Center for Vaccine Research, UNITED STATES

## Abstract

**Objective:**

To compare the time from entry into care for HIV infection until combination antiretroviral therapy (cART) initiation between migrants and non migrants in France, excluding late access to care.

**Methods:**

Antiretroviral-naïve HIV-1-infected individuals newly enrolled in the FHDH cohort between 2002–2010, with CD4 cell counts &gt;200/μL and no previous or current AIDS events were included. In three baseline CD4 cell count strata (200–349, 350-499, ≥500/μL), we examined the crude time until cART initiation within three years after enrolment according to geographic origin, and multivariable hazard ratios according to geographic origin, gender and HIV-transmission group, with adjustment for baseline age, enrolment period, region of care, plasma viral load, and HBV/HBC coinfection.

**Results:**

Among 13338 individuals, 9605 (72.1%) were French natives (FRA), 2873 (21.4%) were migrants from sub-Saharan Africa/non-French West Indies (SSA/NFW), and 860 (6.5%) were migrants from other countries. Kaplan-Meier probabilities of cART initiation were significantly lower in SSA/NFW than in FRA individuals throughout the study period, regardless of the baseline CD4 stratum. After adjustment, the likelihood of cART initiation was respectively 15% (95%CI, 1–28) and 20% (95%CI, 2–38) lower in SSA/NFW men than in FRA men who had sex with men (MSM) in the 350-499 and ≥500 CD4 strata, while no difference was observed between other migrant groups and FRA MSM.

**Conclusion:**

SSA/NFW migrant men living in France with CD4 &gt;350/μL at entry into care are more likely to begin cART later than FRA MSM, despite free access to treatment. Administrative delays in obtaining healthcare coverage do not appear to be responsible.

## Introduction

Although migrants represent 8.4% of the whole population living in France with 5.3 million of persons [1], heterosexually acquired HIV infection in migrants accounted for 38% of the 6372 new diagnoses of HIV infection in 2012 [2]. Heterosexual female migrants (1466 individuals, 23.0%) and heterosexual male migrants (960 individuals, 15.0%) were the second and third largest groups among the new diagnoses of HIV infection, after men who have sex with men (MSM), who accounted for 2648 new diagnoses (42.0%) [2]. Migrants also represented 46% of new AIDS diagnoses in 2003–2010 [3]. While migrants from sub-Saharan Africa compose only 13% of the immigrant population in France [1], they represent 67.4% of HIV-infected migrants [2].

Several studies in high-income countries have shown that migrants are at a higher risk of delayed diagnosis of HIV infection and delayed access to care than other individuals [4,5,6,7]. Furthermore, among people living with HIV engaged in care in France in 2010, fewer migrants received combination antiretroviral therapy (cART) for more than six months and fewer had plasma viral loads (pVL) below 50 cp/mL on treatment when compared to non migrant heterosexuals and MSM [8].

Apart from delayed diagnosis of HIV infection, potential differences in healthcare management between migrants engaged in care at an early stage of HIV infection and their non migrant counterparts have not yet been studied. Following recommendations to increase HIV screening [9] and to expand treatment initiation [10,11], our objective was to compare the time to cART initiation and the types of cART regimens prescribed for migrants and non-migrants without access to care at an advanced stage of HIV disease in the French Hospital Database from 2002 to 2010.

## Materials and Methods

### Individuals

Created in 1989, the FHDH (French Hospital Database on HIV) is a large prospective cohort study of HIV-infected individuals aged at least 15 years receiving care in one of the 70 French participating hospitals; in 2010 it included 50% of HIV-infected persons in care in France [12]. The only enrolment criteria are documented HIV-1 or HIV-2 infection, follow-up in a FHDH participating center, and written informed consent. Data submitted by the participating centres to the data coordinating and analysis centre are anonymised, then encrypted. The FHDH was approved by the institutional ethic committees, Commission Nationale de l’Informatique et des Libertés (CNIL) on 27 November 1991 (Journal Officiel, 17 January 1992).

**Fig 1:**
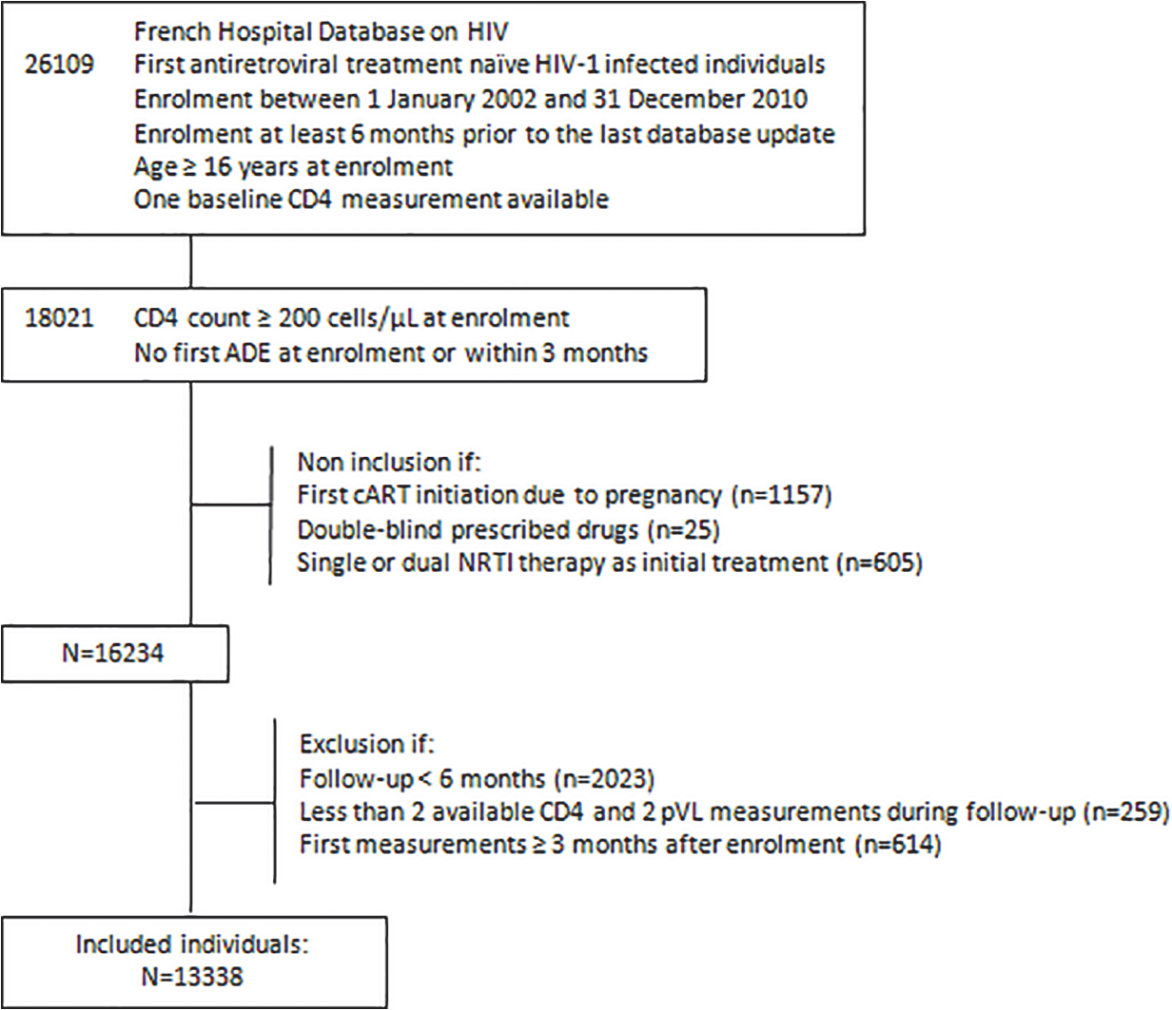
Individuals selection. Abbreviations: ADE, AIDS-defining event; cART, combination antiretroviral therapy; NRTI, Nucleoside Reverse Transcriptase Inhibitor.

The study included antiretroviral treatment-naive HIV-1-infected individuals aged at least 16 years who were newly enrolled in the FHDH between 1 January 2002 and 31 December 2010 with CD4 cell counts above 200/μL and no previous or current AIDS events at enrolment or within the first three months after enrolment. Individuals were excluded if their first cART regimen was initiated for pregnancy, if they participated in a double-blind clinical trial of antiretroviral therapy during their follow-up in the study, or if they had received single or dual NRTI therapy before first-line cART. In order to include only persons who really had the opportunity to begin treatment, those who were lost to follow-up (n = 1995) or died (n = 28) within the first six months after FHDH enrolment or who had no immunovirological assessment during the first three months and fewer than two assessments during follow-up (n = 873) were excluded (Fig. 1).

### Statistical Analysis

Migrant status was based on the United Nations definition: “anyone born and having lived outside France and now residing in France, whatever their nationality and the duration of stay in France” [13]. Regions of origin were subdivided as follows: France (FRA) including French West Indies; sub-Saharan Africa/non-French West Indies (SSA/NFW); and other countries (OTH, mainly North Africa, America, Asia and Europe). Sub-Saharan Africa and non-French West Indies were pooled on the basis of their similar proportion of undocumented migrants.

All antiretroviral combinations that included at least three drugs, or at least two drugs from ritonavir-boosted protease inhibitors (PI/r), non-nucleoside reverse transcriptase inhibitors (NNRTI) and integrase inhibitors (II), or ritonavir-boosted PI monotherapy were considered for the study [10,14]. For descriptive purposes, we distinguished seven groups of cART regimens: regimens with at least two NRTIs plus either an NNRTI, a ritonavir-boosted PI or an integrase inhibitor which are the three currently recommended cART regimens [15,16], regimens with at least three NRTIs or at least two NRTIs plus one non-boosted PI which are two previously recommended cART regimens [17], and two other groups with regimens including at least three drugs or regimens including less than three drugs. These last two groups were individualized because they included potent drug combinations or strategies under investigation, such as boosted PI monotherapy or combinations with one ritonavir-boosted PI plus another potent drug.

Time to first-line cART initiation was calculated from the date of FHDH enrolment to cART initiation, death, the end of follow-up, 31 December 2011, or three years after the beginning of follow-up, whichever occurred first. As the subjects were treatment-naive at enrolment, those who started cART on the day of recruitment were artificially given 1 day of follow-up, as previously described [4].

The Chi2 and Kruskal-Wallis tests were used to compare the characteristics of the individuals at enrolment and at cART initiation, and to evaluate differences in first-line cART regimens across the three regions of origin. Because time to first-line cART initiation varied according to the baseline CD4 cell count, the crude time to cART initiation was assessed, using Kaplan-Meier estimates, according to three baseline CD4 cell count strata (200–349, 350–499, ≥500/μL) and region of origin.

To further explore differences related to geographic origin, gender and HIV transmission group, a combined 7-category variable was created: MSM originating from France (FRA MSM), non homosexual men originating from France (FRA non homosexual men), women originating from France (FRA women), men originating from sub-Saharan Africa/non-French West Indies (SSA/NFW men), women originating from sub-Saharan Africa/non-French West Indies (SSA/NFW women), men originating from other countries (OTH men) and women originating from other countries (OTH women). Multivariable Hazard Ratios (HRs) for first-line cART initiation were determined in Cox models according to geographic origin, gender and HIV transmission group within each baseline CD4 cell count stratum. Age, the enrolment period, the region of care, baseline pVL, and hepatitis B virus antigen and anti-hepatitis C virus antibody status were entered in the multivariable model. Regions of care were defined according to the epidemiology of HIV infection in France, and comprised the Paris area, southern France, French West Indies (Martinique, Guadeloupe, French Guyana), and other regions of metropolitan France plus the Reunion Island [18,19]. Enrolment periods (2002–2005, 2006–2007 and 2008–2010) were chosen according to the evolution of national and international cART initiation guidelines [10,14,17]. We also did a sensitivity analysis by using a multivariable competing risk model taking into account death and censoring occurring prior to cART initiation as a competing risk. All analyses were done with SAS v9.3 software (SAS Institute, Inc, Cary, NC). A p value <0.05 was considered to denote statistical significance.

## Results

### Population

Among 16 234 FHDH individuals meeting the inclusion criteria (11 620 originating from France, 3514 from sub-Saharan Africa/non-French West Indies, 1100 from other countries), 2896 individuals were excluded because of a lack of initial follow-up (Fig. 1). These excluded individuals represented 2015/11 620 FRA individuals (17%), 641/3514 SSA/NFW individuals (18%) and 240/1100 OTH individuals (22%) (p = 0.0008). When comparing these proportions within each CD4 cell count stratum, this difference was only statistically significant in the ≥500 CD4 cell count stratum.

A total of 13 338 individuals were thus included in the study, of whom 9605 (72.1%) were native from FRA, 2873 (21.4%) from SSA/NFW, and 860 (6.5%) from OTH (Table 1). Among the SSA/NFW individuals, 2565 (89.3%) originated from sub-Saharan Africa and 308 (10.7%) from non-French West Indies (mainly Haiti: 80.2%). The SSA individuals were similar to the NFW individuals as regards the sex ratio, HIV transmission group, CD4 cell count, and pVL at enrolment and at cART initiation (data not shown). Among the OTH individuals, 250 (29.1%) originated from Central and South America, 225 (26.2%) from Northern Africa, 154 (17.9%) from Western Europe, 97 (11.3%) from Eastern Europe, 89 (10.4%) from Asia, and 45 (5.1%) from other countries (in the Middle East, North America, Australia and Oceania). MSM accounted for 41.4% of the study population, most of them were from FRA (91.7%) and from OTH (7.1%). Heterosexuals accounted for 48.0% of the overall study population, of whom 54.2% were from FRA, 40.3% from SSA/NFW and 5.5% from OTH. SSA/NFW individuals were slightly younger than FRA and OTH individuals (p<0.0001), with respective median (IQR) ages of 34 (28–41), 36 (30–43) and 35 (29–43) years at FHDH enrolment. The main region of care was the Paris area, particularly for migrants. Migrants, especially those from SSA/NFW, had a lower median baseline CD4 cell count than non migrants (p<0.0001).

**Table 1:** Characteristics of the individuals at enrolment and at cART initiation, N = 13338.

	**All individuals**	**FRA** ^**a**^	**SSA/NFW** ^**b**^	**OTH** ^**c**^	**p**
	**N = 13338**	**N = 9605**	**N = 2873**	**N = 860**	
**At enrolment**					
**Gender and HIV transmission group**					
MSM	5515 (41.4)	5058 (52.7)	68 (2.4)	389 (45.2)	
Heterosexual men	2795 (21.0)	1599 (16.6)	1008 (35.1)	188 (21.9)	
Heterosexual women	3606 (27.0)	1873 (19.5)	1569 (54.6)	164 (19.1)	
IDU men	280 (2.1)	227 (2.4)	6 (0.2)	47 (5.5)	
IDU women	86 (0.6)	74 (0.8)	3 (0.1)	9 (1.0)	
Other men	694 (5.2)	554 (5.7)	90 (3.1)	50 (5.8)	
Other women	362 (2.7)	220 (2.3)	129 (4.5)	13 (1.5)	<0.0001
**Age [years]**					
<30	3712 (27.8)	2519 (26.2)	935 (32.5)	258 (30.0)	
30–39	5124 (38.4)	3669 (38.2)	1140 (39.7)	315 (36.6)	
40–49	2948 (22.1)	2221 (23.1)	546 (19.0)	181 (21.1)	
≥50	1554 (11.7)	1196 (12.5)	252 (8.8)	106 (12.3)	<0.0001
**Enrolment period**					
2002–2005	6902 (51.8)	4882 (50.8)	1604 (55.8)	416 (48.4)	
2006–2007	2927 (21.9)	2100 (21.9)	635 (22.1)	192 (22.3)	
2008–2010	3509 (26.3)	2623 (27.3)	634 (22.1)	252 (29.3)	<0.0001
**Region of care**					
Paris area	7360 (55.2)	4799 (50.0)	1996 (69.5)	565 (65.7)	
Southern France	1193 (8.9)	1023 (10.6)	90 (3.1)	80 (9.3)	
Other/Reunion island	4122 (30.9)	3397 (35.4)	577 (20.1)	148 (17.2)	
French West Indies	663 (5.0)	386 (4.0)	210 (7.3)	67 (7.8)	<0.0001
**CD4 cell count [/μL]**	431 (316–588)	450 (330–610)	375 (282–508)	435 (313–590)	<0.0001
**Plasma viral Load [log10 copies/mL]**	4.42 (3.78–4.95)	4.47 (3.85–5.00)	4.24 (3.58–4.78)	4.46 (3.89–4.92)	<0.0001
**Hepatitis B antigen**					
Negative	9999 (75.0)	6875 (71.6)	2407 (83.8)	717 (83.4)	
Positive	835 (6.3)	316 (3.3)	231 (8.0)	36 (4.2)	
Unknown	2756 (20.6)	2414 (25.1)	235 (8.2)	107 (12.4)	<0.0001
**Hepatitis C antibodies**					
Negative	9966 (74.7)	6767 (70.4)	2533 (88.2)	666 (77.4)	
Positive	835 (6.3)	633 (6.6)	103 (3.6)	99 (11.5)	
Unknown	2537 (19.0)	2205 (23.0)	237 (8.2)	95 (11.1)	<0.0001
**At cART initiation**	**N = 8543**	**N = 6156**	**N = 1845**	**N = 542**	
**CD4 cell count [/μL]**	309 (246–405	319 (252–419)	284 (229–361)	295 (235–394)	<0.0001
**Plasma viral Load [log10 copies/mL]**	4.65 (3.95–5.14)	4.70 (4.00–5.17)	4.50 (3.80–5.00)	4.77 (4.19–5.12)	<0.0001
**AIDS status**	145 (1.7)	88 (1.4)	45 (2.4)	12 (2.2)	0.0082

Data are counts (proportions) and medians (interquartile range).

^a^ Including French West Indies.

^b^ Including non-French West Indies.

^c^ Including Northern Africa, Asia, Oceania, Australia, North America, Central and South America, Middle East, Western Europe and Eastern Europe.

Abbreviations: FRA, French natives; SSA, sub-Saharan Africa; NFW, non-French West Indies; OTH, Other regions of the world; MSM, Men who have sex with men; IDU, Injecting drug user, cART, combination antiretroviral therapy;

During a total follow-up of 18 736 person-years, 8543 individuals started first-line cART and 34 died before cART initiation. Median follow-up from the date of enrolment to the end of the study period whether or not cART was initiated, was 36 months in each group of individuals, whatever the level of the combined variable (geographic origin, gender and HIV transmission group). Migrants had lower CD4 cell counts at cART initiation than FRA individuals (p<0.0001). Among individuals who started cART, a first AIDS event occurred before cART initiation in 145 cases, consisting of tuberculosis in 41 cases (28.3%), Kaposi’s sarcoma in 29 (20.0%), esophageal candidiasis in 17 (11.7%), non-Hodgkin’s lymphoma in 16 (11.0%) and *Pneumocystis jirovecii* pneumonia in 14 (9.7%).

### Antiretroviral regimens

The first-line cARTs are shown in Table 2. Currently recommended cART regimens, i.e. at least two NRTIs plus either an NNRTI, a ritonavir-boosted PI or an integrase inhibitor, were initiated in respectively 4192 (49.1%), 2856 (33.4%) and 185 (2.2%) individuals. Regimens including at least two NRTIs plus an NNRTI or a ritonavir-boosted PI were the most prescribed regimens, whatever the individuals’ origin. Previously recommended cART regimens, i.e. at least three NRTIs or at least two NRTIs plus one non-boosted PI, were initiated in 945 individuals (11.1%). Other cARTs were initiated slightly more frequently in FRA individuals (4.8%) than in SSA/NFW (3.0%) and OTH individuals (3.0%).

**Table 2:** First-line cART regimens started during follow-up, N = 8543.

	All individuals	FRA	SSA/NFW	OTH	p
	N = 8543	N = 6156	N = 1845	N = 542	
**Initial cART**					
≥2 NRTIs + PI/r	4192 (49.1)	2986 (48.5)	951 (51.6)	255 (47.1)	
≥2 NRTIs + NNRTI	2856 (33.4)	1995 (32.4)	657 (35.6)	204 (37.6)	
≥2 NRTIs + II	185 (2.2)	161 (2.6)	13 (0.7)	11 (2.0)	
≥3 NRTIs	585 (6.9)	436 (7.1)	106 (5.7)	43 (7.9)	
≥2 NRTIs + PI	360 (4.2)	284 (4.6)	63 (3.4)	13 (2.4)	
≥3 other drugs^a^	128 (1.5)	104 (1.7)	15 (0.8)	9 (1.7)	
<3 drugs^b^	237 (2.7)	190 (3.1)	40 (2.2)	7 (1.3)	<0.0001

Data are counts (proportions).

^a^ Mainly PI/r + II or PI/r + NNRTI or 2 PIs/r.

^b^ Mainly PI/r monotherapy or 1 II + 1 PI/r or 1 PI/r + 1 NNRTI.

Abbreviations: FRA, French natives; SSA, sub-Saharan Africa; NFW, non-French West Indies; OTH, Other regions of the world; MSM, Men who have sex with men; cART, combination antiretroviral therapy; NRTI, Nucleoside Reverse Transcriptase Inhibitor; PI/r, ritonavir-boosted protease inhibitor; NNRTI, Non-nucleoside Reverse Transcriptase Inhibitor; II, Integrase inhibitor; PI, Protease Inhibitor.

### cART initiation

**Fig 2:**
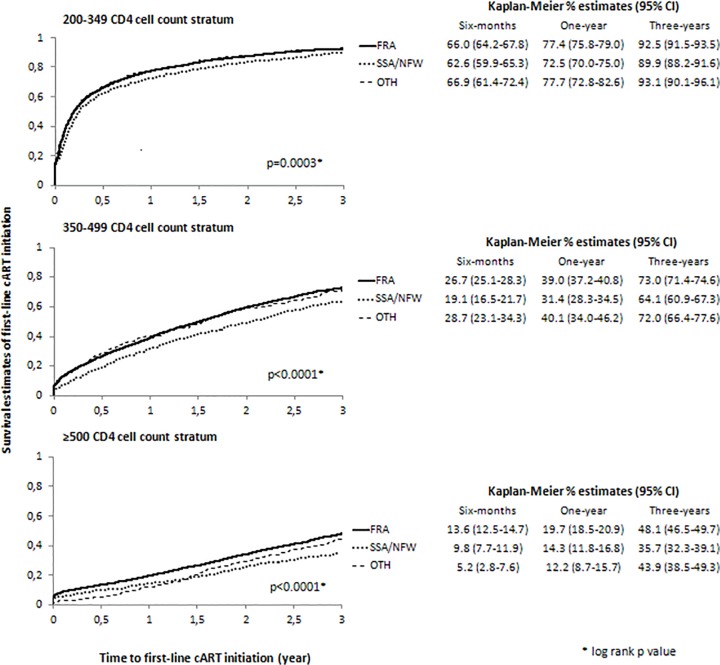
Kaplan-Meier survival estimates of first-line cART initiation by geographic origin and CD4 cell counts [/μL] at enrolment. Abbreviations: cART, combination antiretroviral therapy; FRA, French natives; SSA, sub-Saharan Africa; NFW, non-French West Indies; OTH, Other regions of the world.

As shown in Fig. 2, in univariable analyses of each CD4 cell count stratum, time to cART initiation differed significantly across the three individual origins, SSA/NFW individuals starting cART later than individuals of other origins. For example, in the 350–499 CD4 cell count stratum the one-year probability of cART initiation was 39.0% (37.2–40.8) in FRA individuals, 31.4% (28.3–34.5) in SSA/NFW individuals and 40.1% (34.0–46.2) in OTH individuals.

### 200-349 CD4 cell count stratum

**Fig 3:**
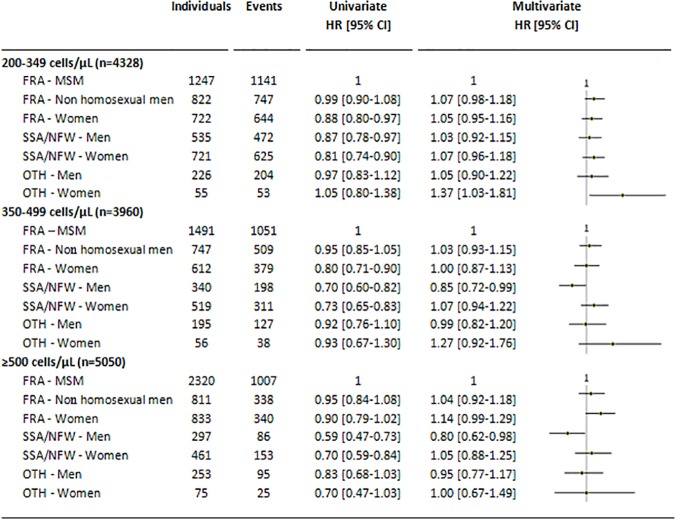
Univariate and multivariate Hazard Ratios (HRs) for cART initiation according to geographic origin, gender and HIV transmission group in baseline CD4 cell count strata (Cox model). * HR adjusted for age at enrolment, enrolment period, region of care, pVL at enrolment, and hepatitis B antigen and hepatitis C antibody status; Abbreviations: cART, Combination antiretroviral therapy; FRA, French natives; SSA, sub-Saharan Africa; NFW, non-French West Indies; OTH, Other regions of the world; MSM, Men who have sex with men.

When differences in cART initiation were analyzed by geographic origin, gender and HIV transmission group (Fig. 3), FRA women and SSA/NFW men and women had a lower likelihood of cART initiation than FRA MSM in univariable analysis (p = 0.0003). After adjustment, the only significant difference was that OTH women were 37% (95%CI, 3–81) more likely than FRA MSM to start cART during the first three years of follow-up.

### 350-499 CD4 cell count stratum

Univariable analysis gave similar results in this stratum. In adjusted analyses, SSA/NFW men were 15% (95%CI, 1–28) less likely than FRA MSM to start cART during their first three years of follow-up. Median times to cART initiation were 71 weeks for FRA MSM and 113 weeks for SSA/NFW men; the respective 25^th^ percentile times to cART initiation were 21 weeks and 38 weeks.

### ≥500 CD4 cell count stratum

In univariable analysis, SSA/NFW men and women were less likely than FRA MSM to start cART. In multivariable analysis the adjusted likelihood of cART initiation was 20% (95%CI, 2–38) lower in SSA/NFW men than in FRA MSM, while the difference was no longer significant between SSA/NFW women and FRA MSM. Interestingly, although the difference was not significant, FRA women were 14% (95%CI, -1–29) more likely than FRA MSM to start cART. Follow-up was too short to estimate median times to cART initiation. The 25^th^ percentile times to cART initiation were 68 weeks for FRA MSM and 109 weeks for SSA/NFW men. In all three CD4 cell count strata the results were unaffected when individuals originating from non-French West Indies were excluded from the SSA/NFW group or when competing risk methods were used (data not shown).

## Discussion

The likelihood of cART initiation was 15% (95%CI, 1–28) lower among migrant men originating from sub-Saharan Africa or non-French West Indies than among MSM originating from France when the baseline CD4 cell count was 350–499/μL, and 20% (95%CI, 2–38) lower when the baseline CD4 cell count was ≥500/μL. No difference between the other migrant groups versus MSM originating from France was observed in these two strata. In the subpopulation of patients with baseline CD4 cell counts of 200–349/μL, migrant women originating from other countries than sub-Saharan Africa or non-French West Indies had a higher adjusted Hazard Ratio for cART initiation than MSM originating from France.

The FHDH cohort, because of its very large size and extensive follow-up, gave us the opportunity to assess differences in the timing of cART initiation between migrants and non migrants with early-stage HIV infection in a high income country, according to their geographic origin, gender, HIV transmission group, and baseline CD4 cell count. In addition, in France, initial evaluation, first antiretroviral therapy initiation and annual exminations and antiretroviral renewals are required in an hospital context by physicians expert in HIV infection care [20]. Therefore, this hospital database is a very suitable way to study those differences in cART initiation. One potential limitation of this study could be difference in losses to follow-up between migrants and non migrants. After adjustment for age, the enrolment period, the region of care, pVL, hepatitis B antigen and hepatitis C antibody status, all migrant men whatever their geographic origin and FRA non homosexual men had a higher risk of being lost to follow-up than MSM originating from France in the CD4 cell count stratum 350–499/μL, and migrant men from other countries than sub-Saharan Africa/non-French West Indies had a higher risk of being lost to follow-up than MSM originating from France in the CD4 cell count stratum ≥500/μL (data not shown). The higher rates of loss to follow-up among the two groups of migrant men could have led to overestimation of the frequency of cART initiation in these groups in the two highest CD4 cell count strata, and would thus have only diminished the observed difference in the time to cART initiation. Similar results were obtained when individuals who were excluded because their follow-up was too short to start treatment, if required by their clinical status, were included in multivariable models (data not shown). We also excluded pregnant women from the study, and it is possible that some missing data on pregnancy status led to an overestimation of the Hazard Ratio for first-line cART initiation among women. It is also conceivable that some women with high baseline CD4 cell counts were prescribed first-line cART when they expressed their intention to become pregnant. This might explain the higher relative hazard of cART initiation among women migrants from other countries than sub-Saharan Africa/non-French West Indies with baseline CD4 cell counts between 200 and 350/μL, and the lack of difference in the risk of cART initiation between women originating from sub-Saharan Africa/non-French West Indies and MSM from France in all three CD4 cell count strata, contrasting with the difference observed between migrant men from sub-Saharan Africa/non-French West Indies and MSM from France.

Despite multivariable analyses taking several potential confounding factors into account, residual confounders cannot be ruled out. Within each baseline CD4 stratum, counts were slightly lower in migrants than in MSM born in France, but the results were unaffected when multivariable analyses included baseline CD4 cell counts within each stratum (data not shown). The proportion of individuals enrolled within six months of primary infection was higher among MSM from France (735 individuals, 14.5%) than among individuals originating from sub-Saharan Africa/non-French West Indies (38 individuals, 3.2%). This was particularly apparent in the ≥500 CD4 stratum, with respective proportions of 359/2320 (15.5%) and 13/297 (4.4%); cART was started in respectively 209/359 (3-year Kaplan-Meier probability: 63.8%; 95%CI, 58.9–68.8) and 9/13 (3-year KM probability: 71.2%; 95%CI, 46.5–95.8) of these individuals. When the models were also adjusted for inclusion during *versus* after the first six months of primary infection, similar results were obtained showing that primary infection was not the main driver of the observed difference (data not shown).

Previous studies of the comparative timing of treatment initiation considered either the overall HIV-infected population whatever the CD4 cell count [4] or the only individuals with a known date of seroconversion [21]. They showed no major differences in the timing of cART initiation between individuals originating from sub-Saharan Africa and non migrants after adjustment for the CD4 cell count at HIV diagnosis. We excluded individuals with CD4 cell count below 200/μL and/or a current or previous AIDS event at HIV diagnosis, as these individuals are likely to start cART rapidly in France, irrespective of their administrative status, financial resources and healthcare coverage. Indeed, antiretroviral therapy is provided free of charge in France for all individuals requiring urgent treatment, and for all other individuals able to demonstrate they have been living in France for more than 3 months [22]. Given the large proportion of patients presenting with low CD4 cell counts, excluding these patients from our analyses explains the difference observed between our results and results found by others [4].

One important finding in our study is the lower likelihood of cART initiation among men from sub-Saharan Africa/non-French West Indies who had high CD4 cell counts (above 350/μL) at FHDH enrolment, an observation probably reflecting the respect of treatment initiation guidelines [10,11] for all individuals with CD4 cell counts between 200 and 350/μL, regardless of their geographic origin, gender and HIV transmission group [23,24], whereas geographic origin may play a role in the decision to treat persons with higher counts. In France, in addition to later HIV screening [8,25,26] and later access to care [6], migrant men from sub-Saharan Africa and non-French West Indies also start cART later than MSM from France when diagnosed at an early stage of HIV infection, despite free universal healthcare. In the 350–499/μL CD4 cell count stratum, the median time to cART initiation was 71 weeks for MSM originating from France and 113 weeks for men originating from sub-Saharan Africa/non-French West Indies, and the respective 25^th^ percentile times to cART initiation were 21 and 38 weeks. In the ≥500/μL CD4 cell count stratum, the 25^th^ percentile times to cART initiation were 68 weeks for MSM originating from France and 109 weeks for men originating from sub-Saharan Africa/non-French West Indies. These times are longer than the three to four months needed to obtain healthcare coverage after entry into care.

Proportions of currently recommended cART (ie at least 2 NRTIs plus either a ritonavir-boosted PI, a NNRTI or an integrase inhibitor) were higher in migrants (87.6%) than in non migrants (83.5%) unlike in other study [4]. Investigational strategies are more often prescribed in people originating from France, and it is conceivable that MSM from France, more volunteer to HIV screening than migrants from sub-Saharan Africa and being more aware of HIV/AIDS care and treatments [26], may ask for newer antiretroviral combinations which could differ with the current recommended treatments [22].

The later cART initiation in male migrants originating from sub-Saharan Africa/non-French West Indies with relatively high CD4 cell counts requires further studies to determine the respective roles of patients and their physicians’ decisions. It is possible, for instance, that physicians delay cART initiation because they anticipate poor adherence to treatment related to socio-economic deprivation, with a risk of lower efficacy and selection of resistant viruses. Alternatively, migrants may hesitate to start treatment because of a lack of knowledge of HIV infection, misconceptions of the HIV transmission risks, or fear of stigmatization, due to socioeconomic, cultural or language barriers [27]. A better understanding of the factors underlying the delayed cART initiation in these populations could help to design appropriate interventions, in the context of new French national guidelines recommending antiretroviral therapy for all HIV-infected individuals regardless of their CD4 cell count, and promoting “combined prevention” based on behavioral measures, testing strategies and antiretroviral therapy, i.e. treatment as prevention [11,28].

## Supporting Information

S1 Appendix Clinical Epidemiology Group of the FHDH-ANRS CO4 cohort. (DOC)
