# Supplementary material for: Later cART Initiation in Migrant Men from Sub-Saharan Africa without Advanced HIV Disease in France
Source: PLoS One. 2015 Mar 3;10(3):e0118492. doi: 10.1371/journal.pone.0118492 (PMC4348541; doi:10.1371/journal.pone.0118492)
Supplement: S1 Appendix — (DOC) [file pone.0118492.s001.doc]

**Appendix**

**Clinical Epidemiology Group of the FHDH-ANRS CO4 cohort**

- **Scientific committee**: S Abgrall, F Barin, E Billaud, F Boué, L Boyer, A Cabié, F Caby, A Canestri, D Costagliola, L Cotte, P De Truchis, X Duval, C Duvivier, P Enel, J Gasnault, C Gaud, J Gilquin, S Grabar, MA Khuong, O Launay, A Mahamat, M Mary-Krause, S Matheron, G Melica, H Melliez, JL Meynard, J Pavie, L Piroth, I Poizot-Martin, C Pradier, J Reynes, E Rouveix, A Simon, P Tattevin, H Tissot-Dupont, Treatment and research inter-associative group TRT-5, JP Viard
- **COREVIH coordinating center**: French Ministry of Health (C Bronnec, D Martin), Technical Hospitalization Information Agency, ATIH (N Jacquemet).
- **Statistical analysis center**: U1136 INSERM et UPMC (D Costagliola (principal investigator), S Abgrall, S Grabar, M Guiguet, S Lang, L Lièvre, M Mary-Krause, H Selinger-Leneman), INSERM Transfert (JM Lacombe, V Potard)
- **COREVIH:** **Paris area**: ***Corevih Ile de France Centre*** (Paris-GH Pitié‑Salpétrière; Paris-Hôpital Saint-Antoine; Paris-Hôpital Tenon), ***Corevih Ile de France Est*** (Bobigny-Hôpital Avicenne; Bondy-Hôpital Jean Verdier; Paris-GH Lariboisière-Fernand Widal; Paris-Hôpital Saint-Louis), ***Corevih Ile de France Nord*** (Paris-Hôpital Bichat-Claude Bernard; St Denis-Hôpital Delafontaine), ***Corevih Ile de France Ouest*** (Argenteuil-CH Victor Dupouy; Boulogne Billancourt-Hôpital Ambroise Paré; Colombes-Hôpital Louis Mourier; Garches-Hôpital Raymond Poincaré; Le Chesnay-Hôpital André Mignot; Mantes La Jolie-CH François Quesnay; Meulan-CHI de Meulan les Mureaux; Nanterre-Hôpital Max Fourestier; Poissy-CHI de Poissy; St Germain en Laye-CHI de St Germain en Laye; Suresnes-Hôpital Foch), ***Corevih Ile de France Sud*** (Clamart-Hôpital Antoine Béclère; Créteil-Hôpital Henri Mondor; Kremlin Bicêtre-Hôpital de Bicêtre; Paris-GH Tarnier-Cochin; Paris-Hôpital Européen Georges Pompidou; Paris-Hôpital Hôtel Dieu; Paris-Hôpital Necker adultes).

**Outside the Paris area**: ***Corevih Alsace*** (CH de Mulhouse; CHRU de Strasbourg), ***Corevih de l’Arc Alpin*** (CHU de Grenoble), ***Corevih Auvergne-Loire*** (CHU de Clermont-Ferrand; CHRU de Saint-Etienne); ***Corevih Basse-Normandie*** (CHRU de Caen), ***Corevih Bourgogne*** (CHRU de Dijon), ***Corevih Bretagne*** (CHU de Rennes), ***Corevih Centre*** (CHRU de Tours), ***Corevih Franche-Comté*** (CH de Belfort; CHRU de Besançon); ***Corevih Haute-Normandie*** (CHRU de Rouen), ***Corevih Languedoc-Roussillon*** (CHU de Montpellier; CHG de Nîmes), ***Corevih Lorraine*** (Nancy-Hôpital de Brabois; CHRU de Reims), ***Corevih de Midi-Pyrénées*** (Toulouse-CHU Purpan; Toulouse-CHU Rangueil; Toulouse-Hôpital la Grave), ***Corevih Nord-Pas de Calais*** (CH de Tourcoing), ***Corevih PACA Est*** (Nice-Hôpital Archet 1; CHG Antibes-Juan les Pins ; Hôpital de Fréjus-St Raphaël ; Grasse-Centre Hospitalier Clavary), ***Corevih PACA Ouest*** (Marseille-Hôpital de la Conception; Marseille-Hôpital Nord; Marseille-Hôpital Sainte-Marguerite; Marseille-Centre pénitentiaire des Baumettes; CHG d’Aix-En-Provence; CH d’Arles; CH d’Avignon; CH de Digne Les Bains; CH de Gap; CH de Martigues; CHI de Toulon), ***Corevih Pays de la Loire*** (CHRU de Nantes), ***Corevih de la Vallée du Rhône*** (Lyon-Hôpital de la Croix-Rousse; Lyon-Hôpital Edouard Herriot).

**Overseas**: ***Corevih Guadeloupe*** (CHRU de Pointe-à-Pitre; CH Saint-Martin), ***Corevih Guyane*** (CHG de Cayenne), ***Corevih Martinique*** (CHRU de Fort-de-France), ***Corevih de La Réunion*** (St Denis-CHD Félix Guyon).
